# Rainbow trout in seasonal environments: phenotypic trade‐offs across a gradient in winter duration

**DOI:** 10.1002/ece3.1636

**Published:** 2015-10-08

**Authors:** Ellen V. Lea, Jonathan A. Mee, John R. Post, Sean M. Rogers, Stephanie Mogensen

**Affiliations:** ^1^ Department of Biological SciencesUniversity of Calgary2500 University Dr. NWCalgaryAlbertaCanadaT2N 1N4; ^2^ Ellen V. Lea, Fisheries and Oceans Canada1 Arctic Road PO Box 1871InuvikNorthwest TerritoriesCandaX0E 0T0

**Keywords:** Bioenergetics, elevation gradient, energy deficit, life‐history, metabolic demands, overwinter survival, trade‐offs

## Abstract

Survival through periods of resource scarcity depends on the balance between metabolic demands and energy storage. The opposing effects of predation and starvation mortality are predicted to result in trade‐offs between traits that optimize fitness during periods of resource plenty (e.g., during the growing season) and those that optimize fitness during periods of resource scarcity (e.g., during the winter). We conducted a common environment experiment with two genetically distinct strains of rainbow trout to investigate trade‐offs due to (1) the balance of growth and predation risk related to foraging rate during the growing season and (2) the allocation of energy to body size prior to the winter. Fry (age 0) from both strains were stocked into replicate natural lakes at low and high elevation that differed in winter duration (i.e., ice cover) by 59 days. Overwinter survival was lowest in the high‐elevation lakes for both strains. Activity rate and growth rate were highest at high elevation, but growing season survival did not differ between strains or between environments. Hence, we did not observe a trade‐off between growth and predation risk related to foraging rate. Growth rate also differed significantly between the strains across both environments, which suggests that growth rate is involved in local adaptation. There was not, however, a difference between strains or between environments in energy storage. Hence, we did not observe a trade‐off between growth and storage. Our findings suggest that intrinsic metabolic rate, which affects a trade‐off between growth rate and overwinter survival, may influence local adaptation in organisms that experience particularly harsh winter conditions (e.g., extended periods trapped beneath the ice in high‐elevation lakes) in some parts of their range.

## Introduction

Mid‐ to high latitudes are typically characterized by a high degree of environmental heterogeneity. Seasonal environmental fluctuations (e.g., winter versus summer) vary tremendously across a gradient in latitude or elevation (e.g., long and harsh versus short and mild winters). Populations that occupy such environmental gradients experience different annual cycles of resource availability and predator abundance, which influence behavioral, physiological, and ecological processes with important fitness consequences (McNamara and Houston 2008; Varpe et al. 2009).

During the growing season, a higher foraging rate increases an organism's consumption rate (Askey et al. 2007), but higher foraging rate also increases exposure to predation risk (Houston et al. 1993; Mangel and Stamps 2001). Hence, in many species, high rates of consumption are associated with a high risk of predation (Ali et al. 2003; Biro et al. 2004a). In some species (fishes in particular), predation risk is often highest for small individuals (Post and Evans 1989; Persson et al. 1996; Parkinson et al. 2004), and faster growth is, in such cases, a means to escape predation at an earlier life stage and increase survival to later life stages. But, foraging at a higher rate to achieve faster growth comes with an increased risk of predation during the growing season. Alternatively, for a given foraging rate, an elevated metabolic rate can allow faster growth due to an increased capacity for rapid biosynthesis (Stoks et al. 2006; Scharf et al. 2009) or food processing (Bochdansky et al. 2005; Stevens and Devlin 2005; Millidine et al. 2009; Dupont‐Prinet et al. 2010). But, high metabolic rate and food processing ability are known to reduce starvation endurance during periods of energy deficit (Afik and Karasov 1995; Cant et al. 1996; Bochdansky et al. 2005; Stoks et al. 2006; Millidine et al. 2009; Scharf et al. 2009; Dupont‐Prinet et al. 2010; Killen et al. 2011).

Winter is typically a period of energy deficit, and overwinter survival depends on the balance between metabolic demands and energy storage (Chippindale et al. 1996; Schultz and Conover 1997, 1999; Gotthard 2001). Many organisms, including both ectotherms (Derickson 1976; Fitzpatrick 1976; Arts and Evans 1991; Dratnal et al. 1993; Post and Parkinson 2001; Hurst and Conover 2003; Dupont‐Prinet et al. 2010) and endotherms (White and West 1977; Carey et al. 1978), allocate energy to storage in order to fuel overwinter metabolic demands. Body size is an important component of the energetic equation governing overwinter survival because, proportional to their mass, small individuals have higher metabolic rates and lower energy reserves relative to larger individuals (Post and Parkinson 2001; Hurst and Conover 2003; Dupont‐Prinet et al. 2010).

**Figure 1:**
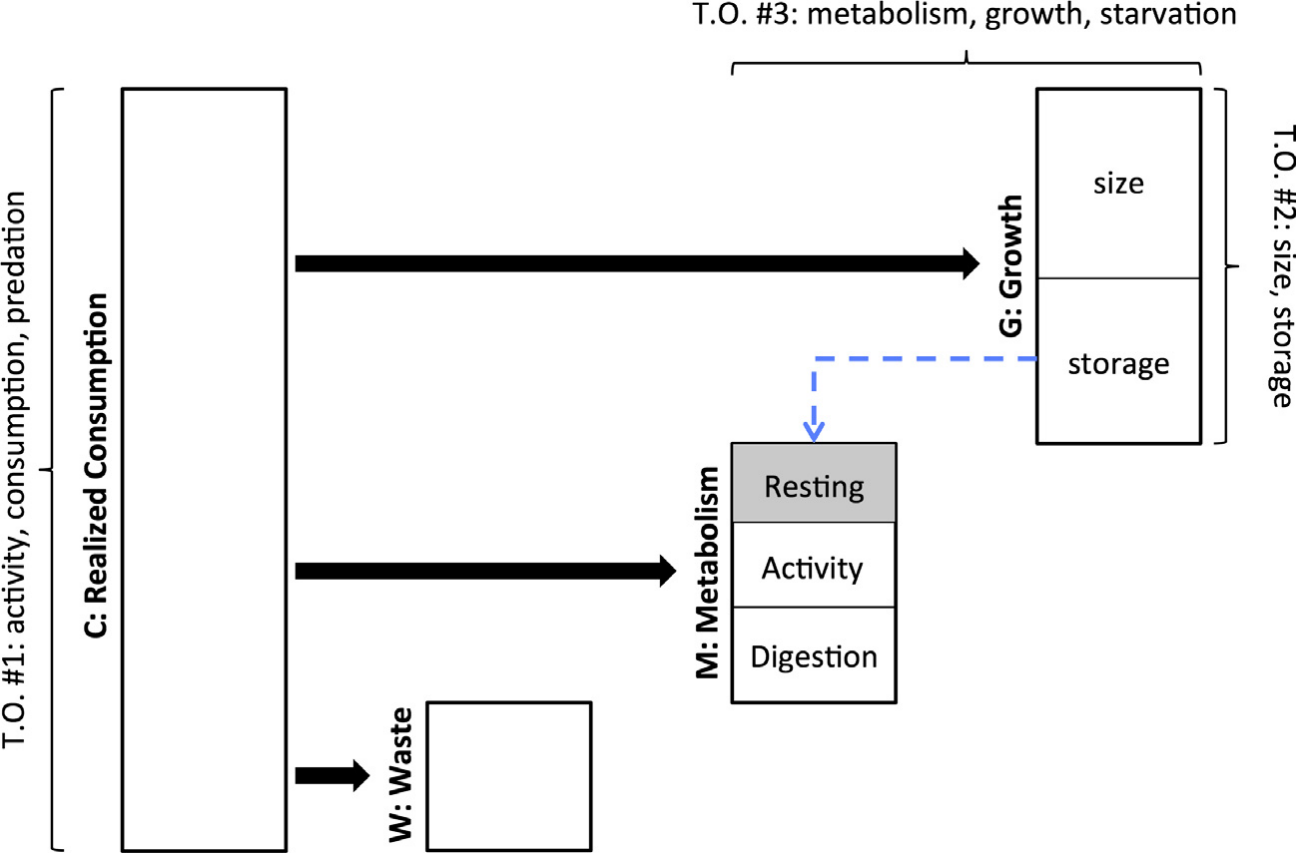
Simplified depiction of the bioenergetics model that predicts several trade‐offs, including (T.O. #1) the trade‐off between consumption rate and predation risk mediated by activity rate, (T.O. #2) the trade‐off between size and storage, (T.O. #3) the trade‐off between growth rate and starvation resistance mediated by metabolic rate, and (T.O. #4) the trade‐off between consumption rate and consumption efficiency. The latter two trade‐offs were not directly evaluated in the present study. The heights of the rectangles in this graphic represent an amount of energy that can vary with respect to allocation, whereas the widths are arbitrary. The gray box represents metabolic demand for energy during winter that can only be supplied by stored energy (dashed line) in the absence of consumption.

The full suite of behavioral, physiological, and ecological traits for organisms distributed in heterogeneous seasonal environments, therefore, emerges from a balance among a complex series of bioenergetic trade‐offs within and between seasons. These trade‐offs are due to (1) the balance of growth and predation risk during the growing season (Houston et al. 1993; Ali et al. 2003; Biro et al. 2004a), (2) the allocation of energy to growth (i.e., size) and storage prior to the winter (Chippindale et al. 1996; Gotthard 2001; Post and Parkinson 2001; Dupont‐Prinet et al. 2010), and (3) the metabolic demands of rapid growth and food processing ability during the growing season and decreased tolerance of starvation in the winter. A simplified bioenergetics model (modified from Hanson et al. 1997; Nisbet et al. 2012) helps to demonstrate these trade‐offs (Fig. 1):

C=G+M+W,

where *C* is consumption rate (i.e., the realized rate at which food is consumed), *G* is growth rate (i.e., the rate of conversion of energy from food into either size or storage), *M* is metabolic rate, and *W* is waste. Metabolic rate includes both respiration rate and specific dynamic action (a.k.a., the cost of digestion). Respiration rate depends on a combination of body size and temperature and includes a component related to activity (i.e., higher activity rate results in high respiration rate) and a resting component.

The trade‐off between consumption rate and predation risk (T.O. #1, Fig. 1) is not purely a bioenergetic trade‐off, but is a classic example of how bioenergetic and ecological processes interact (Houston et al. 1993). The trade‐off between size and storage (T.O. # 2, Fig. 1) has frequently been cited as the primary mechanism driving adaptive differences between populations distributed across altitudinal or latitudinal gradients (Schultz and Conover 1997; Tracy 1999; Gentle and Gosler 2001; Brodin 2007; Macleod et al. 2008; Berg et al. 2009; Jonsson et al. 2009; Finstad et al. 2010; Takahashi and Pauley 2010; Mogensen and Post 2012). The trade‐off between growth and starvation mediated by metabolic rate (T.O. #3, Fig. 1) has been demonstrated for terrestrial vertebrate populations distributed across altitudinal or latitudinal gradients (Stockhoff 1991; Gotthard et al. 1994; Scharf et al. 2009), but direct evidence for fishes is lacking (but see Wieser et al. 1992; Arnott et al. 2006; Killen et al. 2007). Even with no activity and very low temperatures, respiration imposes a metabolic demand for energy (gray box in Fig. 1) that can only be supplied by stored energy when consumption ceases during the winter (dashed line in Fig. 1). The magnitude of this winter metabolic demand (and the resulting starvation risk) will increase with higher metabolic rates, but higher metabolic rates during the growing season are associated with more gut tissue, higher activity rates, more rapid conversion of food into energy, and consequently, higher consumption and faster growth (Cant et al. 1996; Bochdansky et al. 2005; Stoks et al. 2006; Scharf et al. 2009; Dupont‐Prinet et al. 2010; Killen et al. 2011). Hence, for T.O. #3 to exist, metabolic rate during periods of starvation must be correlated with metabolic rate during periods of growth (e.g., from eq. 2: *f*(T), a and b must be independent of season, but can vary among populations and/or individuals). A correlation of metabolic rate between seasons, however, has never been directly evaluated, although the prediction that individuals with the highest metabolic rates during the growing season will also have the highest metabolic rates in the winter (i.e., the rank among individuals should be consistent between seasons) is supported by the observation that metabolic rate is repeatable for individuals (Nespolo and Franco 2007).

Juvenile rainbow trout (*Oncorhynchus mykiss*) are an excellent candidate for the study of bioenergetic trade‐offs because they avoid the complication of allocation to reproduction, their growing season survival is strongly linked to body size and predation risk (Post and Evans 1989; Persson et al. 1996; Sogard 1997; Post et al. 1998; Post and Parkinson 2001; Parkinson et al. 2004; Biro et al. 2006), and their overwinter survival is dependent on a minimum threshold quantity of energy storage (Biro et al. 2004b, 2005; Mogensen and Post 2012). Furthermore, populations of rainbow trout are distributed throughout the mid‐latitudes in western North America, across a large altitudinal gradient with a corresponding gradient in the duration of winter. Rainbow trout distributed across heterogeneous environments vary in fitness‐related traits, which suggests that persistence of rainbow trout populations in different environments depends on differences in fitness‐related traits (Taylor 1991; Carvalho 1993; McCusker et al. 2000; Keeley et al. 2005, 2007; Taylor et al. 2011). However, the extent to which the persistence of rainbow trout populations across a gradient in winter duration is related to variation in energy allocation strategies and/or bioenergetic trade‐offs is unknown, as is the extent to which variation in life‐history traits (e.g., growth, energy storage, foraging rate, metabolic rate) across this gradient may be attributed to differences between particular strains or genotypes.

In this study, we conducted a common environment experiment in high‐ and low‐elevation lakes with juvenile (age 0) rainbow trout from two populations from two regions with different phylogeographic histories (Tamkee et al. 2010) to investigate phenotypic variation between environments that differ in winter duration. Juveniles from these populations were stocked into replicate natural lakes at low and high elevation. We investigated the existence of a trade‐off between foraging activity and predation risk (T.O. #1) by measuring variability in activity rate and growing season survival across low‐ and high‐elevation environments, and we investigated the existence of a trade‐off between body size and storage (T.O. #2) by measuring growth rate and lipid concentration across environments. We hypothesized that foraging, predation, and energy allocation trade‐offs would result in population‐ and environment‐specific differences in growth rate, energy storage, and foraging activity. We predicted that high foraging rate should be associated with low growing season survival and that high growth rate should be associated with low energy storage (and *vice versa*). We tested for consistent differences between populations (i.e., genotypic effects) across environments, for consistent differences between environments across genotypes (i.e., environmental effects), and for genotype‐by‐environment interactions. We also indirectly evaluated the role of a trade‐off between growth and starvation (T.O. #3) by comparing theoretical starvation thresholds, based on a range of hypothetical metabolic rates, to observed variation in energy storage across environments and genotypes. The use of replicate whole natural lakes, which allow natural environmental variability to influence the experimental response to our treatment of interest (i.e., winter duration), strengthens our ability make inferences about the strategies employed by rainbow trout and other species that live in seasonal environments to cope with environmental heterogeneity.

## Methods

### Rainbow trout populations

The FFSBC (Freshwater Fisheries Society of British Columbia) manages several strains of rainbow trout (one domesticated and several wild strains) for stocking across the province. The wild strains are maintained in a series of broodstock lakes that receive progeny from wild sources. Every spring, spawners are collected from these broodstock lakes, and fertilized eggs are transported to FFSBC hatcheries for rearing. Two wild strains were chosen from the FFSBC system for these experiments: Blackwater Dragon 2N (hereafter referred to as Blackwater) and Pennask Premier 2N (hereafter referred to as Pennask). The Blackwater strain is originally from the Blackwater River (elevation: 700–900 m) in the upper Fraser River drainage, located 80 km northwest of Quesnel, BC, and is maintained in nearby Dragon Lake (elevation: 598 m). The Pennask strain is originally from Pennask Lake (elevation: 1426 m) in the Thompson River drainage, east of Merritt, BC, and is maintained in Premier Lake (elevation: 877 m), 70 km north of Cranbrook, BC. For our study, we used fish raised at the Vancouver Island Trout Hatchery (VITH, located near Duncan, BC), the Clearwater Trout Hatchery (CWTH, near Clearwater, BC), and the Fraser Valley Trout Hatchery (FVTH, in Abbotsford, BC).

A sample of each strain (*n* = 30) was collected for genetic analysis to confirm genetic differentiation between strains. DNA was extracted from fin clips using a standard phenol: chloroform extraction technique. Fifteen microsatellite loci were assayed for each sample (Table 1). Assay conditions were the same as those described in Tamkee et al. (2010), except fluorescently labeled primers were used instead of ^32^P labeled primers, and PCRs were not multiplexed. The PCR product from each reaction was diluted to a concentration of 20 ng/μL, and alleles were separated on an Applied Biosystems 3730 DNA Analyzer (Applied Biosystems, Foster City, CA 94404 USA). Microsatellite allele fragment length polymorphism was scored using GeneMapper v3.7 (Applied Biosystems, Foster City, CA 94404 USA). Basic genetic diversity metrics (Table 1) were calculated with the HIERFSTAT package for R (Goudet 2005). LD (Linkage disequilibrium) and deviations from HWE (Hardy–Weinberg equilibrium) were investigated using the pegas package for R (Paradis 2010). The degree of genetic differentiation between Blackwater and Pennask strains was determined by calculating Weir and Cockerham's *F*_ST_ in the HIERFSTAT package. The significance of the *F*_ST_ value was determined via the 95% confidence interval derived from bootstrapped variance components, as suggested by Weir and Cockerham (1984), calculated using the boot.vc command (with 1000 bootstraps) in the HIERFSTAT package.

**Table 1:** Genetic diversity of Blackwater and Pennask rainbow trout at the 15 microsatellite loci analyzed in this study. Asterisks indicate loci used in the calculation of the overall *F*_ST_. Allelic richness is the rarified allelic counts, with the number of alleles rarified down to the number of individuals genotyped times 2

	Ho			Hs			Allelic richness			*F*_IS_			
Locus	BW	PN	Total	BW	PN	Total	BW	PN	Total	BW	PN	Total	*F*_ST_
Occ16*	0.27	0.36	0.31	0.46	0.46	0.46	2.00	2.00	2.00	0.41	0.22	0.31	−0.03
Occ34	0.05	0.10	0.07	0.05	0.09	0.07	1.52	1.78	1.94	0.00	−0.03	−0.02	−0.01
Occ42*	0.05	0.19	0.12	0.05	0.18	0.11	1.52	1.96	1.99	0.00	−0.08	−0.06	0.01
Okia3	0.73	0.63	0.68	0.81	0.57	0.69	6.00	4.35	9.84	0.11	−0.10	0.02	0.16
Omy77*	1.00	0.67	0.83	0.86	0.73	0.79	7.76	7.10	9.34	−0.17	0.08	−0.05	0.03
Oneu8	0.52	0.71	0.62	0.77	0.75	0.76	6.26	5.36	7.50	0.32	0.05	0.19	0.05
Oneu14*	0.68	0.15	0.42	0.50	0.19	0.35	2.00	2.90	3.28	−0.36	0.22	−0.20	0.16
Ots3	0.35	0.52	0.44	0.69	0.65	0.67	4.35	4.03	4.85	0.50	0.19	0.35	0.04
Ots4*	0.90	0.79	0.84	0.68	0.71	0.70	4.10	5.90	6.78	−0.33	−0.10	−0.21	0.03
Ots100*	0.78	0.81	0.79	0.75	0.85	0.80	5.51	6.77	7.91	−0.03	0.05	0.01	0.02
Ots103	0.22	0.26	0.24	0.52	0.52	0.52	2.00	2.00	2.00	0.58	0.49	0.53	−0.02
Ots107	0.89	0.63	0.76	0.79	0.53	0.66	7.90	4.57	11.44	−0.13	−0.19	−0.16	0.16
Ssa85*	0.75	0.75	0.75	0.73	0.54	0.64	4.00	2.69	4.89	−0.02	−0.39	−0.18	0.04
Ssa197*	0.75	0.19	0.47	0.51	0.28	0.39	2.00	2.00	2.00	−0.48	0.32	−0.20	0.15
Ssa456*	0.57	0.21	0.39	0.46	0.20	0.33	3.42	2.51	3.97	−0.25	−0.07	−0.20	0.04
Overall			0.52			0.53			5.32			0.02	0.09

### Experimental lakes and fish stocking

We used natural lakes to conduct a replicated common environment experiment over two growing seasons (2008 and 2009) using the Blackwater and Pennask strains. Three lakes located southeast of Merritt, BC, were selected to comprise a “low‐elevation” treatment: Noname Lake (elevation: 981 m), Cigar Lake (1000 m), and Smoke Lake (1001 m). Two lakes located near Bonaparte Provincial Park northeast of Kamloops, BC, were selected to comprise a “high‐elevation” treatment: Pantano Lake (1479 m) and Spook Lake (1498 m). In the summer of 2009 (prior to stocking in that year), Pantano Lake was divided into two experimental units (Big Pantano and Little Pantano) using a combination of rebar and mesh to create a barrier impermeable to fish movement. Mark–recapture sampling of differently marked fish on either side of this barrier in October of 2009 confirmed the impermeability of the barrier to fish. We recorded temperature and duration of ice cover in all of these lakes from July 2008 through October 2009 using HOBO U22 Water Temp Pro v2 temperature loggers installed at a depth of 2 m. Initially, two temperature loggers were installed per lake (one at a northern location and one at a southern location), but several data loggers were lost to vandalism, and records are absent for some periods. Several other biotic and abiotic characteristics (which are peripheral to our hypothesized effects of winter duration on phenotypic trade‐offs) of all lakes were recorded, and these are described in detail in supplementary material.

In an attempt to equalize the density of conspecific predators and competitors among the study lakes, we gill‐netted all the lakes to remove existing rainbow trout in the spring prior to initiating the experiments in 2008, and we stocked yearling (age 1) Pennask rainbow trout from the FFSBC Summerland Trout Hatchery (Summerland, BC). The presence of conspecific predators imposes behavioral, growth, and survival constraints on juvenile rainbow trout that were necessary for ecological realism in our experiments (Post et al. 1998, 1999; Landry et al. 1999; Biro et al. 2003a,b; Askey et al. 2007). Yearling Pennask fish (age 1, nonexperimental fish) from the FFSBC Summerland Trout Hatchery were stocked in June 2008 at a density of 150 fish per hectare after being marked with a unique fin clip to distinguish them from any fish remaining in the lakes that may have escaped gill netting. Smoke Lake was restocked with Pennask yearlings in June 2009 because complete anoxic winterkill eliminated all fish in this lake in the winter of 2008–2009. We used Pennask yearlings (as opposed to a mixture of Pennask and Blackwater) due to constraints associated with rearing capacity at the FFSBC hatcheries.

Each experimental fish (age 0 fry) was given a pelvic fin clip for strain identification: left pelvic fin for Blackwater and right pelvic fin for Pennask. To reduce unwanted mortality among our experimental fish due to fin clipping and to increase the accuracy of fin clips, we waited to stock our experimental lakes until fry had reached a minimum size (≥0.6 g). During rearing at the hatchery, water temperature and food rations were adjusted to match the size of the two strains at the time of stocking. Replicate batch samples of fry (>200 individuals per sample) were weighed and counted. On 29 August ^h^ 2008, Pennask fry (mean weight: 0.85 g) and Blackwater fry (0.75 g) from two hatcheries (CWTH and VITH) were stocked into Pantano Lake, Spook Lake, Cigar Lake, Noname Lake, and Smoke Lake at a density of 2255 fry per hectare. On 26 August 2009, Pennask fry (0.65 g) and Blackwater fry (0.66 g) from FVTH were stocked into Little Pantano, Big Pantano, Cigar Lake, and Smoke Lake at a density of 2800 fry per hectare. We used fewer experimental lakes in 2009 than in 2008 because of the logistics and time constraints involved with conducting mark–recapture population estimates in 2009.

### Survival

Our hypotheses and predictions are based on the assumption that the risk of overwinter starvation is higher in high‐elevation lakes than in low‐elevation lakes, and therefore, that the balance of mortality risks during the winter and during the growing season (i.e., risk of starvation or predation, respectively) is different in low and high‐elevation lakes. In order to investigate this assumption, we used Petersen mark–recapture methods (Seber 1982; Krebs 1989) to obtain population estimates in the spring of 2009. Immediately following ice‐off in the spring of 2009 (early May in the low‐elevation lakes, early June in the high‐elevation lakes), for five to eight consecutive netting nights, fry were captured with fyke nets, marked with a dorsal or ventral clip on the caudal fin, and then released. Five to ten days later, fish were lethally sampled using gill nets (with mesh sizes ranging from 13 to 89 mm, set at depths ranging from 1 to 6 m, as per Post et al. 1999; Askey et al. 2007) and fyke nets (hoop diameter of 0.5 m, mesh diameter of 6 mm, set perpendicular to shore in littoral and deep littoral areas). Lethal sampling consisted of five consecutive nights across all lakes. In each lake, nets were set at midday and had a soaking time of 18–24 hours, after which captured fish were removed and nets were reset in a new location. An estimate of population size at the time of marking (${\hat{N}}_{t}$) was obtained using the equation:

$${\hat{N}}_{t}=\frac{\left(M+1)(C+1\right)}{R+1}-1,$$

where *M* is the number of individuals marked in the first sample, *C* is the number of individuals captured in the second sample, and *R* is the number of marked individuals in the second sample (Seber 1982). In order to obtain an estimate of survival $\left({\hat{S}}_{t}\right)$ for each strain in both environments, we calculated this value $\left({\hat{N}}_{t}\right)$ for both strains in each lake and used the equation ${\hat{S}}_{t}={\hat{N}}_{t}/N_{0}$, where *N*_0_ is the number of fry (of a particular strain) initially stocked into a particular lake in the summer of 2008 minus the number of fish removed during fall sampling in 2008.

This is a coarse measure of “overwinter” survival (and, hence, mortality) because we do not have a population estimate for the fall of 2008, and consequently, we cannot separate growing season mortality (prior to ice over in the fall of 2008) from mortality that actually occurred during the winter under the ice. We were, however, able to determine whether 2009 growing season survival differed between environments and/or between strains by conducting a population estimate in the fall of 2009 using the same methodology as in the spring. This comparison of growing season survival among strains and environments allowed us to interpret any differences between strains or environments in “overwinter” survival as differences in actual overwinter survival (i.e., beneath the ice) or as differences in growing season survival. For our estimate of growing season survival, ${\hat{N}}_{t}$ was calculated in the same manner as for overwinter survival (except sampling occurred during the first two weeks of October 2009), and *N*_0_ was the number of fry (of a particular strain) initially stocked into a particular lake in the summer of 2009. This fall 2009 mark–recapture study also confirmed that the barrier between Little Pantano and Big Pantano was impermeable to fish movement because no Little Pantano fish (ventral clip on the caudal fin) were caught in Big Pantano Lake (dorsal clip on the caudal fin), or vice versa.

### Growth

We estimated growth rate over the period between summer stocking and fall capture in 2008 and 2009. Fish were lethally sampled using fyke nets (hoop diameter of 0.5 m, mesh diameter of 6 mm, set perpendicular to shore in littoral and deep littoral areas) before the lakes iced‐over in the fall of 2008 and 2009. In both years, sampling occurred starting on October 2 or 8 in the high‐elevation lakes and low‐elevation lakes, respectively. Sampling consisted of five consecutive nights in each lake. Nets were set at midday and had a soaking time of 18–24 hours, after which captured fish were removed and nets were reset in a new location in that lake. For all individuals captured, we recorded fork length (to the nearest 1 mm) and wet mass (to the nearest 0.01 g). An estimate of mass‐specific growth rate (% per day) for each fish was calculated using the following formula (after Myrick and Cech 2000):

$$\mathrm{MSGR}\left(\%\;\;\mathrm{body}\;\;\mathrm{weight}\;\;\mathrm{per}\;\;\mathrm{day}\right)=\frac{W_{2}-W_{1}}{0.5\left(W_{1}+W_{2}\right)\mathrm{days}}\times 100\%,$$

where *W*_1_ is the strain‐specific mean weight at the time of stocking, and *W*_2_ is the weight of each fish at the time of capture. We estimated growth relative to calendar days and relative to growing degree days (gdd). To determine what constitutes one degree day for rainbow trout growth, we used the growth rate and temperature data from Myrick and Cech (2000) and Hokanson et al. (1977) to predict the curvilinear relationship between temperature and growth rate for age 0 rainbow trout. Then, using the average temperature over the growing season in high and low‐elevation lakes, we adjusted the number of days of growth to reflect the gdd, where growth over one gdd at any given temperature is equivalent to growth over 1 day at the optimum temperature (17.6°C, calculated from Hokanson et al. 1977; Myrick and Cech 2000).

### Energy storage

Fish primarily use stored lipids to fuel overwinter metabolism (Shuter and Post 1990; Biro et al. 2004b). We investigated prewinter energy storage based on an analysis of fall lipid concentration. Throughout our fall sampling (described above), a subset of individuals sampled for growth measurements were placed on ice immediately after capture then frozen at the earliest opportunity. In the laboratory, lipid analysis was conducted on approximately 25 samples of each strain–lake–year combination. Each sample consisted of a pair of length‐matched fish (±2 mm) to allow for sufficient dry weight (0.5 g) for the lipid extraction methodology. Pairs were chosen using stratified random sampling by length so that pairs would represent the full size range for each strain–lake–year combination. Fish were thawed, measured, and weighed, and then they were dried at 50°C for 96 h in a drying oven on an aluminum foil boat. The dried fish were weighed and then finely ground using a mortar and pestle until reduced to a homogeneous powder. Lipids were extracted from these dried samples using the methanol and chloroform procedure from Folch et al. (1957), which is described in detail by Post and Parkinson (2001) and Biro et al. (2004b, 2005). The extracted lipid was weighed to the nearest 0.0001 g, and this mass represented the amount of lipid per 0.5 g of dry fish tissue. We expect an average coefficient of variation for this estimate of lipid concentration of less than 6% for repeated measurements taken from the same sample (Post and Parkinson 2001).

There is a threshold lipid concentration below which winter metabolic demands will outstrip energy storage leading to starvation, and this threshold is dependent on size (due to the allometry of specific metabolic rate), winter duration, and intrinsic (i.e., size‐independent) variation in metabolic rate (Biro et al. 2004b; Mogensen and Post 2012). Larger fish tend not only to have greater lipid reserves than smaller fish, but also that larger fish have a lower metabolic rates than smaller fish (Shuter and Post 1990; Schultz and Conover 1999; Post and Parkinson 2001). In the absence of any data on metabolic rate in these fishes, we used previously published models and data relating to *O. mykiss* (both rainbow trout and Steelhead Trout) metabolism and lipid storage (Rand et al. 1993; Hanson et al. 1997; Myrick and Cech 2000; Biro et al. 2004b; Mogensen and Post 2012) to hypothesize a range of threshold lipid concentrations given observed winter duration, water temperature, and fish size. These thresholds were based on the intrinsic metabolic rate from Mogensen and Post (2012). In particular, we took the estimated intercept of the linear relationship between mass and respiration rate for rainbow trout at 0°C (11.16623; from Mogensen and Post 2012), as well as intercepts 25% and 50% higher and lower, to generate a range of threshold curves against which to compare actual lipid concentrations.

### Activity

We estimated foraging activity using a measurement of catchability for each strain in all lakes in the fall of 2009. Using the data from our fall 2009 mark–recapture (described above), our measurement of catchability (*q*) was based on the number of marked individuals recaptured in fyke nets (*R*) relative to the sampling effort with fyke nets at the time of recapture during fall lethal sampling (*F*) and the total number of marked fish in the population (*M*) according to Ricker's (1975) model: *R*/*F* = *qM*. We used fyke net recaptures only in this calculation because, unlike gill nets, fyke nets are not size selective. In order to be caught in these nets, fish must be active. We assumed that activity associated with being caught in a fyke net was representative of foraging activity (i.e., that all movement by age 0 rainbow trout was motivated by foraging).

### Statistical analyses

There is a direct correspondence between sources of phenotypic variation (i.e., effects of genotype, environment, and genotype‐by‐environment interaction) and the interpretation of main and interaction effects in an analysis of variance (Pigliucci 2001). As such, ANOVA has been a major method in the analysis of experiments investigating environmental and genetic effects (Lewontin 1974; Westcott 1986). For all of the phenotypes measured in this study (i.e., survival, growth, lipid concentration, and catchability), we used AN(C)OVA to test for an association with strain, environment, and a strain‐by‐environment interaction. When data were available for multiple years, we used mixed effects modeling to account for random variation between years (i.e., we included year as a random effect). Also, when we were able to use individual fish as a unit of replication (i.e., for growth and lipid data), lake was also included as a random effect (nested within year where data were available for multiple years). To account for the allometric relationship between size and lipid concentration, we included log‐transformed wet mass as a covariate in an ANCOVA, and we log‐transformed lipid concentration to obtain a linear relationship in our model (Biro et al. 2004b). All statistical analyses and data plotting were conducted in R (R Development Core Team 2010). Mixed effects modeling was conducted using the nlme package (Pinheiro et al. 2014), which uses denominator degrees of freedom (denDF) corresponding to the classical decomposition of degrees of freedom in ANOVA designs (Pinheiro and Bates 2000).

## Results

### Rainbow trout strains and experimental lakes

Analysis of microsatellite variation at fifteen loci (Table 1) revealed significant LD for one pair of loci (Occ34 and Occ42). In our analysis of genetic differentiation, we used only one of these two linked loci (results are shown for the inclusion of Occ42, but *F*_ST_ estimates were identical when Occ34 was included instead). Significant deviations from HWE were found for five loci (Okia3, Ots3, Ots103, Ots107, and Oneu8), and we dropped all these loci from our analysis of genetic differentiation. Based on nine loci showing no LD or deviations from HWE, we found significant genetic differentiation between fish sampled from the Blackwater and Pennask strains (*F*_ST_ = 0.092; 95% confidence interval = 0.051–0.204; Table 1).

**Figure 2:**
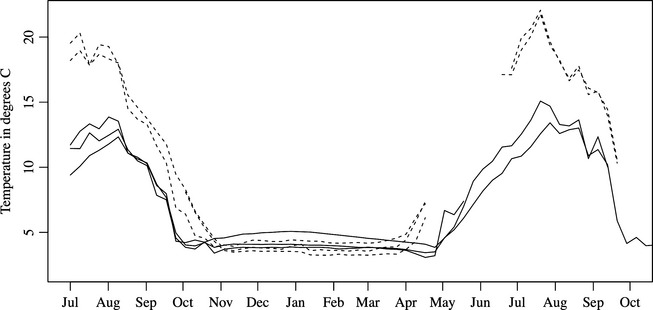
Temperature variation in low‐elevation (dashed lines) and high‐elevation (solid lines) experimental lakes from July 2008 through October 2009. Temperature readings were taken every four hours. The lines have been smoothed with a LOWESS function (Cleveland 1979, 1981).

Temperature differences between low‐elevation and high‐elevation lakes were as expected (Fig. 2). Low‐elevation lakes reached a maximum summer temperature of 20–22°C, while high‐elevation lakes reached a maximum summer temperature of 12–15°C. Low‐elevation lakes were iced‐over for 145 days, while high‐elevation lakes were iced‐over for 204 days. Additional details on lakes are included in supplementary material.

### Survival

**Figure 3:**
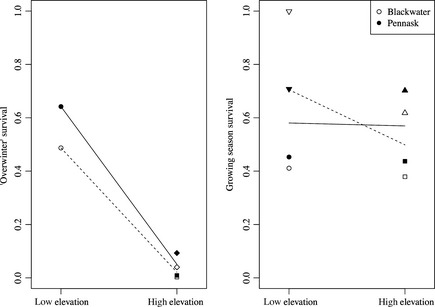
Survival through to the spring of 2009 for juvenile (age 0) rainbow trout stocked in the summer of 2008 (left panel), and survival through to the fall of 2009 for juvenile rainbow trout stocked in the summer of 2009 (right panel). Points indicate survival rates for Blackwater (open points) and Pennask (filled points) fish in each experimental lake (circles: Cigar Lake; upside down triangles: Smoke Lake; diamonds: Spook Lake; squares: Pantano Lake or Big Pantano Lake; triangles: Little Pantano Lake). Lines show estimated reaction norms for Blackwater (dashed lines, open symbols) and Pennask (solid lines, filled symbols) strains calculated from the average survival in each environment (across all lakes).

Two low‐elevation lakes, Smoke Lake and Noname Lake, experienced complete anoxic winterkill. No fish of any kind were caught in these lakes during spring sampling in 2009. Anoxic winterkill can occur when relatively shallow, high‐productivity lakes are covered in ice, preventing atmospheric oxygen from mixing with the water while respiration continues to use up oxygen in the water, resulting in asphyxiation (Dodds 2002). We excluded these lakes from our analysis of overwinter survival because winterkill is a different mechanism of mortality (typically resulting in an all‐or‐nothing pattern of mortality) than overwinter starvation. We clipped a total of 1,099 fish in the spring of 2009, and 303 of 2,377 fish caught during recapture sampling were clipped. Note that we measured survival proportion $\left({\hat{S}}_{t}\right)$ for each lake, and we analyzed these proportions (with lake as the unit of replication) with the expectation of normally distributed values between 0 and 1 (Rogers et al. 2014). There was a significant effect of environment on survival of fish stocked in 2008 through to spring of 2009 (“overwinter” survival, Fig. 3, Table 2). “Overwinter” survival was higher in low‐elevation lakes than in high‐elevation lakes (denDF = 2, *F = *177, *P = *0.0056). There was no significant effect of strain on “overwinter” survival (denDF = 2, *F = *3.63, *P = *0.197), and the strain‐by‐environment interaction was also nonsignificant (denDF = 2, *F = *2.50, *P = *0.255). We clipped a total of 4770 fish in the fall of 2009, and 957 of 5000 fish caught during recapture sampling were clipped. There were no significant differences in 2009 growing season survival (Fig. 3, Table 3) between environments (denDF = 4, *F = *0.352, *P = *0.585) or strains (denDF = 4, *F = *0.0213, *P = *0.891), nor was there a significant environment‐by‐strain interaction (denDF = 4, *F = *0.284, *P = *0.622).

**Table 2:** ANOVA table for analysis of overwinter survival data (response variable: overwinter survival proportion, ${\hat{S}}_{t}$)

Effects:	denDF	*F*	*P*
Environment	2	177.04	0.0056
Strain	2	3.63	0.1969
Env.‐by‐strain	2	2.50	0.2549

**Table 3:** ANOVA table for analysis of growing season survival data (response variable: growing season survival proportion, ${\hat{S}}_{t}$)

Effects:	denDF	*F*	*P*
Environment	4	0.35	0.5851
Strain	4	0.02	0.8911
Env.‐by‐strain	4	0.28	0.6223

### Growth

In 2008, experimental fish in low‐ and high‐elevation lakes were allowed to grow over a period of 42 and 35 calendar days, respectively. In 2009, experimental fish in low‐ and high‐elevation lakes were allowed to grow over a period of 45 and 38 calendar days, respectively. In 2008, fish in low‐ and high‐elevation lakes experienced mean temperatures of 12.9 and 9.8 degrees C, respectively. In 2009, fish in low‐ and high‐elevation lakes experienced mean temperatures of 15.1 and 11.5 degrees C, respectively. Based on previously published data on the growth of age 0 rainbow trout at different temperatures (Hokanson et al. 1977; Myrick and Cech 2000), we predicted the following relationship between MSGR and temperature: MSGR_*t*_ = −2.85 + 0.79(*t*) – 0.022(*t*)^2^. The optimum temperature (for maximum growth rate) estimated from these data was 17.6°C. Using the formula, gdd = days × MSGR_*t*_/MSGR_*t*.*opt*_, where MSGR_*t*.*opt*_ is the predicted growth rate at optimum temperature, we calculated the number of growing degree days experienced by fish stocked at low and high elevation in 2008 to be 36.9 and 23.4, respectively, and in 2009 to be 43.5 and 30.3, respectively.

**Figure 4:**
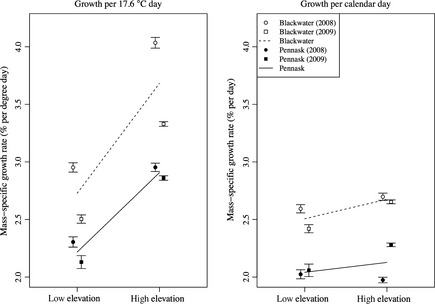
Growth rate of Blackwater (open points) and Pennask (filled points) fish in 2008 (circles) and 2009 (squares). Whiskers show standard error relative to the mean values for each strain, in each environment, in each year (averaged across all experimental lakes). Lines show estimated reaction norms for Blackwater (dashed lines) and Pennask (solid lines) strains calculated from the average growth in each environment (across both years and across all lakes).

During fall sampling, we captured and measured 1124 fish across all lakes in all environments in 2008, and 1962 fish in 2009 (Fig. 4). The maximum mass‐specific growth rate across all fish was 3.83% per day or 5.54% per gdd, and the minimum was −2.59% per day or −3.87% per gdd (negative values are possible because stocking weight was an average from a sample of 200 fish for each strain in each year, while fall weight was measured for each individual fish at the time of capture). The maximum fall weight across all fish was 6.83 g, and the minimum was 0.32 g. There were significant effects of strain and environment on growth rate of fish (Fig. 3, Tables 4 and 5). MSGR was higher in the Blackwater (low elevation) strain than in the Pennask (high elevation) strain when analyze relative to days (denDF = 7, *F = *47.6, *P = *0.0002) or gdd (denDF = 7, *F = *36.6, *P = *0.0005). MSGR was higher in high‐elevation lakes than in low‐elevation lakes when analyzed relative to gdd (denDF = 6, *F = *11.99, *P = *0.0134) but not when analyzed relative to days (denDF = 6, *F = *0.0483, *P = *0.833). There was no significant strain‐by‐environment interaction for MSGR per day (denDF = 7, *F = *0.209, *P = *0.661) or per gdd (denDF = 7, *F = *1.29, *P = *0.293).

**Table 4:** ANOVA table for analysis of growth data (response variable: MSGR per calendar day)

Random Effects:			
Year			
Lake(Year)			
Fixed Effects:	denDF	*F*	*P*
Intercept	3068	732.48	<0.0001
Environment	6	0.05	0.8333
Strain	7	47.64	0.0002
Env.‐by‐strain	7	0.21	0.6612

**Table 5:** ANOVA table for analysis of growth data (response variable: MSGR per gdd)

Random Effects:			
Year			
Lake(Year)			
Fixed Effects:	denDF	*F*	*P*
Intercept	3068	157.73	<0.0001
Environment	6	11.99	0.0134
Strain	7	36.57	0.0005
Env.‐by‐strain	7	1.29	0.2934

### Energy storage

**Figure 5:**
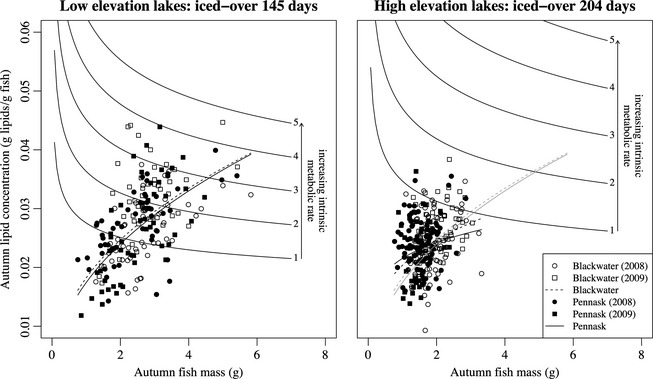
Autumn lipid concentrations in juvenile (age 0) Blackwater and Pennask rainbow trout stocked in high‐ and low‐elevation lakes. Circles represent 2008 data, and squares represent 2009 data, and the regressions between lipid concentration and fish mass are represented by dashed and solid lines of positive slope for Blackwater and Pennask fish, respectively. The gray lines of positive slope in the “high‐elevation lakes” plot are the same lines as in the “low‐elevation lakes” plot – they are repeated for comparison only. The lines of negative slope represent the mass‐specific lipid concentration thresholds below which overwinter survival would be unlikely. These are hypothetical threshold curves are drawn from Mogensen and Post (2012). Line 1 in the “low‐elevation lakes” plot is equivalent to line 1 in the “high‐elevation lakes” plot (and so on) in that they represent the exact same intrinsic metabolic rate.

We analyzed lipid concentration in 257 pairs of fish in 2008, and 209 in 2009, for a total of 466 measurements (Fig. 5). The maximum lipid concentration across all fish was 0.045 g per gram of fish (approximately 0.22 g in a 5.0 g fish), and the minimum was 0.0093 g per gram of fish (approximately 0.16 g in a 1.7 g fish). Lipid concentration was significantly higher in large fish relative to small fish (denDF = 444, *F = *139, *P *< 0.001), and there was a significant interaction between fish size and environment (denDF = 444, *F = *14.7, *P = *0.0001) such that small fish at high elevation had more lipids than the same sized fish at low elevation, and large fish at high elevation had less lipids than the same sized fish at low elevation (Fig. 4, Table 6). There was no significant interaction between size and strain (denDF = 444, *F = *0.010, *P = *0.920). We found no significant effect of strain (denDF = 7, *F = *0.001, *P* = 0.974) or environment (denDF = 6, *F = *0.078, *P* = 0.789) after accounting for the variance in lipid concentration associated with fish size. Similarly, there was no significant interaction between strain and environment (denDF = 7, *F = *0.001, *P = *0.9776).

**Table 6:** ANCOVA table for analysis of lipid concentration data (response variable: log‐transformed lipid concentration). Size, in this table, is shorthand for log‐transformed wet mass

Random Effects:			
Year			
Lake(Year)			
Fixed Effects:	denDF	*F*	*P*
Intercept	444	17,163.37	<0.0001
Size	444	138.67	<0.0001
Environment	6	0.078	0.7888
Strain	7	0.001	0.9743
Size‐by‐strain	444	0.010	0.9204
Size‐by‐env.	444	14.73	0.0001
Env.‐by‐strain	7	0.001	0.9776
Size‐by‐strain‐by‐Env.	444	1.025	0.3120

### Activity

**Figure 6:**
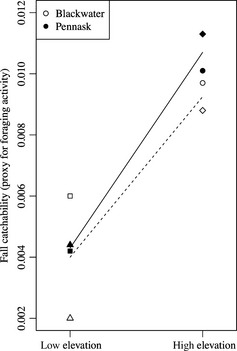
Catchability of juvenile (age 0) rainbow trout estimated in the fall of 2009. Points indicate catchability (*q*) estimates for Blackwater (open points) and Pennask (filled points) fish in each experimental lake (squares: Cigar Lake; triangles: Smoke Lake; diamonds: Little Pantano Lake; circles: Big Pantano Lake). Lines show estimated reaction norms for Blackwater (dashed lines, open symbols) and Pennask (solid lines, filled symbols) strains calculated from the average catchability in each environment.

There was a significant effect of environment on catchability of fish in the fall of 2009 (Fig. 6, Table 7). Note that our measure of catchability (*q*) is a proportion for each lake, and we analyzed these proportions (with lake as the unit of replication) with the expectation of normality for values distributed between 0 and 1 (Rogers et al. 2014). Catchability was higher in high‐elevation lakes than in low‐elevation lakes (denDF = 4, *F = *29.6, *P = *0.0055). There was no significant effect of strain on catchability (denDF = 4, *F = *0.670, *P = *0.459) and the strain‐by‐environment interaction was also nonsignificant (denDF = 4, *F = *0.289, *P = *0.619).

**Table 7:** ANOVA table for analysis of activity (response variable: catchability, *q*)

Effects:	denDF	*F*	*P*
Environment	4	29.68	0.0055
Strain	4	0.67	0.4591
Env.‐by‐strain	4	0.29	0.6192

## Discussion

The opposing effects of predation mortality and starvation mortality on the fitness of different traits result in trade‐offs between traits that optimize fitness during periods of resource plenty (e.g., during the growing season) and periods of resource scarcity (e.g., during the winter). We investigated the existence of trade‐offs between foraging activity and predation risk (T.O. #1, Fig. 1) and between body size and storage (T.O. #2, Fig. 1) in juvenile rainbow trout in a common environment experiment involving two genetically distinct strains in two environments that differed in winter duration by 59 days. We observed much lower overwinter survival in higher elevation environments with longer winters relative to lower elevation environments with shorter winters (Fig. 3). We expected, therefore, that the balance of mortality risk during the growing season and mortality risk during the winter (i.e., risk of predation or starvation, respectively) should be different in low and high‐elevation lakes.

In general, we found little evidence for the existence of trade‐offs between foraging activity and predation risk or between body size and storage. Foraging rates were highest in high‐elevation environments, but, contrary to our predictions, this difference in foraging activity between environments was not associated with a difference in growing season survival. Growth per calendar day did not differ between environments, but fish in high‐elevation lakes did have higher growth per growing degree day, suggesting that these individuals compensate for a shorter growing season by growing faster at high elevation. Contrary to our predictions, however, there was no clear evidence for a trade‐off between growth rate and energy storage in our experiment: There was a difference in growth rate between environments, but no difference in lipid concentration between environments. Furthermore, whereas there was a difference between strains in growth rate, both strains showed the same pattern of energy storage. We did, however, find that the slope of the mass–lipid relationship was shallower for fish in high‐elevation lakes, suggesting that small fish at high elevation had more lipids than the same sized fish at low elevation, and large fish at high elevation had less lipids than the same sized fish at low elevation (i.e., there was a size‐by‐environment interaction). It may be that rapid growth comes at the expense of allocation to storage at high elevation, although no effect of environment (besides the interaction effect) was detected in our experiment (perhaps because the range of fish sizes was limited at high elevation, reducing our power to detect an environmental effect).

At the genotypic level, we did not observe any trade‐off between growth and storage: The Blackwater strain grew faster than the Pennask strain, but there was no genotypic difference in lipid concentration. Other mechanisms are likely responsible for growth rate differences between strains across environments. For example, differences in metabolic rate may be associated with faster growth may indicate faster metabolism (Arendt 1997; Hanson et al. 1997), although a positive correlation between metabolic rate and growth is not consistently detected in field studies of fishes (Alvarez and Nicieza 2005; Burton et al. 2011). Individuals that grow fast during the summer can lose mass faster during the winter and may, therefore, experience higher winter mortality due to starvation (Stockhoff 1991; Gotthard et al. 1994; Scharf et al. 2009). These potential links between starvation risk and growth rate, mediated by metabolic rate, have been clearly demonstrated with terrestrial invertebrates (Stockhoff 1991; Gotthard et al. 1994; Scharf et al. 2009), but direct evidence for fishes is lacking (but see Wieser et al. 1992; Arnott et al. 2006; Killen et al. 2007). We attempted to make the link between starvation risk and metabolic rate using previously published models and data relating to metabolism and lipid storage (Rand et al. 1993; Hanson et al. 1997; Myrick and Cech 2000; Biro et al. 2004b; Mogensen and Post 2012). If all strains had the same intrinsic metabolic rate inferred by Mogensen and Post (2012) in their study of rainbow trout energetics (corresponding to threshold line 3 in Fig. 5), very few individuals (in fact, none of the individuals sampled in the present study) would survive a winter at high elevation. If, however, the reduced growth rates were due to a lower metabolic rate (e.g., corresponding to threshold line 1 in Fig. 5), the number of fish surviving a winter at high elevation would be substantially higher.

The Pennask and Blackwater strains may be adapted to different seasonal effects (e.g., long versus short winters), given that their origins and broodstock lakes differ substantially in elevation. Hence, it is possible that the observed differences in growth rate under a common environment reflect local adaptation to high‐ and low‐elevation environments (perhaps based on differences between strains in metabolic rate). Alternatively, these growth rate differences may reflect the lake versus river origin of these two strains, but there is comparative evidence from fish species distributed across lotic and lentic habitats that latitude (i.e., temperature) is an important factor influencing growth rate, whereas habitat (i.e., river or lake) is not (Blanck and Lamouroux 2007). If we are to fully understand trade‐offs for organisms in seasonal environments, our results point strongly to the need to measure the intrinsic metabolic rates across populations adapted to different seasonal effects.

Our observation of higher growth rate per gdd at high elevation relative to low elevation, and higher growth rate for a putatively low‐elevation‐adapted strain relative to a putatively high‐elevation‐adapted strain, is in contrast with the literature on countergradient variation (Conover and Present 1990; Conover and Schultz 1995; Conover et al. 1997; Schultz and Conover 1997, 1999; Billerbeck et al. 2001; Lankford et al. 2001; Yamahira and Conover 2002; Laugen et al. 2003). Typical models of growth compensation suggest that the response of individuals of a given strain (or population) to decreased temperature should be to decrease growth rate, whereas we see the opposite in our comparison of growth rate across environments. Also, typical models of the evolution of growth compensation suggest that, in order to grow at an acceptable rate in their local environment, strains from cold environments should have higher growth rate at cold temperatures than strains from warm environments and vice versa (i.e., there should be a genotype‐by‐environment interaction), or strains from cold environments should grow faster across all temperatures (i.e., there should be countergradient variation). Again, we observed the opposite effect in our comparison of low‐ and high‐elevation strains. One possible reason for these differences between the results of our study of rainbow trout and previous observations of growth compensation (e.g., in Atlantic silversides, *Menidia menidia*) could be related to the importance of starvation resistance under the ice during the winter in seasonal freshwater environments. It is possible that winter starvation under these conditions is a much stronger factor influencing rainbow trout bioenergetic adaptations than for typical examples of adaptation to thermal gradients in marine or estuarine habitats, as rainbow trout are likely not feeding in the winter (Biro et al. 2004b). This may especially be the case if starvation resistance during the winter and growth rate in the summer is strongly influenced by intrinsic metabolic rate, and if individuals are unable to decouple winter and summer metabolic rates. Further studies of trait variation, and influences on growth rate, among freshwater species across a gradient in winter duration are clearly warranted.

We recognize an important caveat to our inferences regarding environmental effects. We used a relatively small number of lakes for our environmental treatment (three low‐elevation lakes in 2008, two in 2009; two high‐elevation lakes in 2008, one lake split into two in 2009). As is often the case, the high effort and cost associated with conducting whole‐lake experiments in natural lakes limited our ability to include more replicate lakes, although this was balanced by the benefit (in terms of realistic outcomes relevant to natural conditions) of allowing natural environmental variability to influence the behavioral and metabolic responses of individuals in our experiment. Any environmental effects may be confounded by consistent differences between lakes that are arbitrary relative to predictable environmental differences along a gradient in elevation (e.g., in temperature and winter duration). We can identify two potential confounding factors that may be of particular importance. First, the high‐elevation lakes happen to have more gradually sloping shores and higher perimeter to surface area ratios than low‐elevation lakes (see supplemental material), which means high‐elevation lakes have more littoral area (proportional to total area) relative to low‐elevation lakes. This may have been responsible for higher estimated activity rates in high‐elevation lakes because our estimate of activity rate was based on catchability, which is influenced by the amount of littoral surface area (Pierce et al. 2010), but we contend that, given the general appearance of the lakes relative to the variation in lake morphology across the whole landscape (personal observation, JRP), the magnitude of this difference in littoral surface area was not substantial enough to cause the observed effect on activity rates. A second potentially important confounding factor for our interpretation of environmental effects was *Daphnia* concentration, which was higher in high elevation than in low‐elevation lakes (see supplemental material). *Daphnia* are known to be an important forage item for age 0 rainbow trout. Hence, the observed environmental difference in growth (higher in high‐elevation lakes) may have been a result of higher consumption rates in the high‐elevation lakes. We do not, however, know whether consumption rates actually varied in proportion to differences in Daphnia abundance. In order for these differences to be relevant, food availability in the low‐elevation lakes would have had to limit realized consumption to an extent that the relative foraging risks for the growth–mortality trade‐off would have been altered. Given that we observed higher activity in the high‐elevation lakes and that these were all relatively high‐productivity lakes, we contend that this is unlikely. Nonetheless, future studies of environmentally mediated trade‐offs should attempt to compare a higher number of low‐ versus high‐elevation (or warm versus cold) environments while controlling for confounding factors, such as *Daphnia* concentration, that may affect consumption rate.

Common environment experiments conducted in natural ecosystems provide valuable insight into the mechanisms driving variation in life‐history traits across heterogeneous environments. These experiments allow us to distinguish genetically based phenotypic variation from plastic environmental effects on phenotypes and, therefore, give insight into the traits involved in coping with and adapting to differences in winter duration (Bernatchez 2004; Kawecki and Ebert 2004; Rogers and Bernatchez 2005). Identifying trait variation in this context is important in the context of understanding the process of ecological divergence, and in the context of managing natural resources with the goal of preserving biodiversity. Populations of rainbow trout in British Columbia colonized their current distribution following the retreat of glaciers ten to fifteen thousand years ago (Tamkee et al. 2010; Taylor et al. 2011). Understanding how species adapt and persist in postglacial landscapes depends on an understanding of the specific traits involved in trade‐offs across a gradient in winter duration. Identifying the traits involved in ecological divergence across this gradient is, in turn, a prerequisite step in understanding the origins and persistence of biological diversity in heterogeneous landscapes at the most fundamental level.

## Conflict of Interest

None declared.

## Supporting information


**Appendix S1.** Biotic and Abiotic Characteristics of Experimental Lakes.
**Figure S1.** Principal components ordination of eight lake characteristics: average growing season temperature (Avg_Temp_GS), July littoral Daphnia biomass (July_Daph), September littoral Daphnia biomass (Sept_Daph), July littoral zooplankton biomass (July_Zoop), September littoral zooplankton biomass (Sept_Zoop), July littoral macroinvertebrate biomass (July_Macinv), September littoral macroinvertebrate biomass (Sept_Macinv), and effective density of conspecific predators and competitors (PredComp_D).
**Table S1.** Morphometry and location of experimental lakes in south‐central British Columbia.
