# Supplementary material for: Rainbow trout in seasonal environments: phenotypic trade‐offs across a gradient in winter duration
Source: Ecol Evol. 2015 Oct 8;5(21):4778–94. doi: 10.1002/ece3.1636 (PMC4662309; doi:10.1002/ece3.1636)
Supplement: Supplementary file 1 — Appendix S1. Biotic and Abiotic Characteristics of Experimental Lakes. Figure S1. Principal components ordination of eight lake characteristics: average growing season temperature (Avg_Temp_GS), July littoral Daphnia biomass (July_Daph), September littoral Daphnia biomass (Sept_Daph), July littoral zooplankton biomass (July_Zoop), September littoral zooplankton biomass (Sept_Zoop), July littoral macroinvertebrate biomass (July_Macinv), September littoral macroinvertebrate biomass (Sept_Macinv), and effective density of conspecific predators and competitors (PredComp_D). Table S1. Morphometry and location of experimental lakes in south‐central British Columbia. [file ECE3-5-4778-s001.docx]

**Supplementary Material: Biotic and Abiotic Characteristics of Experimental Lakes**

METHODS

*Effective density of conspecific competitors and predators*

To assess the amount of competition and predation experienced by our experimental age-0 fish, we considered the size and abundance of older rainbow trout (i.e., age-1+) in fall of 2008 and 2009. The most appropriate metric for the competitive or predatory effects of these older rainbow trout is known as effective density (Post et al. 1999):

$ED={\sum_{i=1}^{n} L_{i}^{2}}/{Lake area}$,

where *L* is fish length in mm, and *n* is an estimate of the total population of age-1+ fish.

We used Petersen mark-recapture methods (Seber 1982, Krebs 1989) to obtain population estimates. In the first two weeks of October, for five to eight consecutive netting nights, fry were captured with fyke nets, marked with a dorsal or ventral clip on the caudal fin, and then released. Five to ten days later, fish were lethally sampled using gill nets (with mesh sizes ranging from 13 to 89mm, set at depths ranging from 1 to 6m, as per Post et al. 1999, Askey et al. 2007) and fyke nets (hoop diameter of 0.5m, mesh diameter of 6mm, set perpendicular to shore in littoral and deep littoral areas). Lethal sampling consisted of five consecutive nights across all lakes. In each lake, nets were set at midday and had a soaking time of 18-24 hours, after which captured fish were removed and nets were reset in a new location. An estimate of population size at the time of marking $\hat{N}_{t}$ was obtained using the equation

$$\hat{N}_{t}=\frac{(M+1)(C+1)}{R+1}-1$$

where *M* is the number of individuals marked in the first sample, *C* is the number of individuals captured in the second sample, and *R* is the number of marked individuals in the second sample (Seber 1982). All fish were measured (fork length to the nearest 1 mm) at the time of lethal sampling.

*Abundance of zooplankton and macroinvertebrates*

The littoral zone (near-shore to deep-littoral) of each lake was of greatest interest given that this is the primary foraging area for rainbow trout fry, and Daphnidae are the primary source of food for age-0 rainbow trout, accounting for >90% of their diet in similar lake systems (Landry et al. 1999). Zooplankton (including Daphnidae) were sampled mid-day in July and September of 2008 using a 12 L Schindler trap to assess biomass densities in two habitats in each lake: near-shore littoral and deep-littoral (1 m and 2.5 m depth, respectively). This sampling occurred as follows: six near-shore littoral samples at 0.5 m depth and three deep-littoral samples at each of 0.5 and 1.5 m depths. The samples were combined to make a single sample representative of the two habitats within a lake for the day sampled. A subsample of at least 300 individuals was identified to family or genus and counted. The first 30 individuals per taxon were measured to the nearest 0.04 mm with an eyepiece micrometer. Biomass was estimated using density estimates and mean individual mass estimated by genus-specific length versus wet-mass relations from Post (1984). The availability of zooplankton for juvenile rainbow trout is represented as the total zooplankton and total daphnid biomass µg∙L^-1^.

We sampled benthic macroinvertebrates during July 2008 and September 2008 with an artificial substrate method adopted from Benoît et al. (1998). The artificial substrate samplers were designed to be colonized by mobile macroinvertebrates that are common in the diets of larger rainbow trout. These samplers were constructed from 210 x 210 mm ceramic tiles glued with 12 fronds each (9 and 18 cm tall) of plastic aquarium plants designed to simulate foxtail (*Myriophyllum* sp.), a dominant macrophyte found in all experimental lakes. For each sampling period, three sites at approximately 1.5 m total depth were randomly chosen per lake. At each site, 4 tiles were gently laid in a row on the lake bottom approximately 1 m apart. The tiles were laid and retrieved by a snorkeller to minimize sediment disruption. These tiles were left for 96 h, and then collected using a specially fitted Plexiglass box cover with a 500 µm Nitex screen on the top, to allow water to drain while retaining macroinvertebrates within the box. The fronds and tile were washed into the box with a high-pressure water pump and the macroinvertebrates from each tile were preserved in a jar with 70% ethanol. Further detail on the field methodology and the temporal patterns of macroinvertebrate colonization on artificial substrates can be found in Benoît et al. (1998). In the laboratory, invertebrates were sorted, identified to order or family, and then enumerated with the use of a dissecting microscope. Each taxon within a sample was blotted to remove superficial water and then weighed as a group to the nearest 0.0001 g. The average macroinvertebrate biomass of the 4 tiles within a site was then calculated, for a total of 3 sites per sampling event within a lake.

RESULTS

*Effective density of conspecific competitors and predators*

During 2008, the size-structure of age-1+ rainbow trout was fairly consistent among lakes within each environment, with larger fish in the warm lakes (mean length ≈ 270 mm) than in the cold lakes (mean length ≈ 185 mm). Stocked yearlings (age-1) accounted for a majority, if not all of these fish. Cigar Lake also had a small number of older and significantly larger rainbow trout present from experiments in previous years, and these likely imposed a strong consumptive force on fry in this lake in 2008. The size-frequency of older rainbow trout in 2009 was quite different than 2008. Big Pantano Lake and Little Pantano Lake had bimodal size distributions (mean length ≈ 205 mm), and Cigar had many small fish one-year or older (mean length ≈ 195 mm), largely due to the survivors from the 2008 experimental fry cohort. The size distribution of Smoke in 2009 was very similar to 2008 (mean length ≈ 270 mm).

Lakes differed extensively within and between environments in the effective density (∑*L*^2^/lake area, measured in m^2^/ha) of larger trout competitors and predators. In 2008, the effective density of rainbow trout one-year and older was greater in the warm lakes, owing primarily to their larger size rather than their numeric density (Figure S1). For 2009 this same pattern holds between environments. The effective density of larger fish in Little Pantano Lake was substantially less than in Big Pantano Lake, and was higher in Cigar relative to Smoke. Fry from the 2008 cohort in Cigar experienced high survival rates over winter and into spring. Even with attempts to reset the conditions for 2009 with high netting effort in the spring intended to lower these densities, the 2008 experimental fry cohort represented a large component of fish abundance and biomass in the fall of 2009 in Cigar.

*Abundance of zooplankton and macroinvertebrates*

Total littoral zooplankton biomass densities varied among lakes within an environment, but not between environments (Figure S1). Daphnid biomass was particularly low in the littoral region in Cigar and Smoke, and total zooplankton availability was noticeably lower in Cigar than any of the other lakes. Overall littoral biomass densities of Daphnid and total zooplankton were greater in the cold lakes than in the warm lakes (Figure S1). The biomass of benthic macroinvertebrates varied among lakes within an environment and between environments (Figure S1). Amphipods made up a majority of benthic macroinvertebrates in all lakes, both by number and biomass.

LITERATURE CITED

Askey, P. J., J. R. Post, E. A. Parkinson, E. Rivot, A. J. Paul, and P. A. Biro. 2007. Estimation of gillnet efficiency and selectivity across multiple sampling units: A hierarchical Bayesian analysis using mark-recapture data. Fisheries Research **83**: 162-174.

Benoit, H. P., J. R. Post, E. A. Parkinson, and N. T. Johnston. 1998. Colonization by lentic macroinvertebrates: Evaluating colonization processes using artificial substrates and appraising applicability of the technique. Canadian Journal of Fisheries and Aquatic Sciences **55**: 2425-2435.

Krebs, C. J. 1989. Ecological Methodology. Harper & Row, New York.

Landry, F., J. R. Post, and E. A. Parkinson. 1999. Spatial ontogeny of lentic age-0 rainbow trout, Oncorhynchus mykiss: whole-lake manipulations of population size structure. Canadian Journal of Fisheries and Aquatic Sciences **56**: 1916-1928.

Post, J. R. 1984. Planktivorous Fish and the Structure of Pelagic Plankton Communities. M.Sc. thesis. York University, Toronto. 128.

Post, J. R., E. A. Parkinson, and N. T. Johnston. 1999. Density-dependent processes in structured fish populations: Interaction strengths in whole-lake experiments. Ecological Monographs **69**: 155-175.

Seber, G. 1982. The Estimation of Animal Abundance and Related Parameters. 2nd edition. Charles Griffin and Company Ltd., London.

**Figure S1.** Principal components ordination of eight lake characteristics: average growing season temperature (Avg_Temp_GS), July littoral Daphnia biomass (July_Daph), September littoral Daphnia biomass (Sept_Daph), July littoral zooplankton biomass (July_Zoop), September littoral zooplankton biomass (Sept_Zoop), July littoral macroinvertebrate biomass (July_Macinv), September littoral macroinvertebrate biomass (Sept_Macinv), and effective density of conspecific predators and competitors (PredComp_D). PC1 and PC2 summarize 87% of the variation among lakes. Red arrows indicate the relative loadings of the lake characteristics on each PC axis. The scores of each lake for PC1 and PC2 are shown in black (low elevation lakes: Cigar, Noname, Smoke; high elevation lakes: Pantano, Spook).

**Table S1.** Morphometry and location of experimental lakes in south-central British Columbia.

|  | High Elevation Lakes | | | Low Elevation Lakes | | |
| --- | --- | --- | --- | --- | --- | --- |
|  | Big Pantano^1^ | Little Pantano^1^ | Spook | Noname | Cigar | Smoke |
| Surface area (ha) | 1.8 | 1.4 | 4.4 | 1.4 | 3.4 | 2.2 |
| Max. depth (m) | 5.7 | 4 | 5 | 5.5 | 11 | 8 |
| Latitude (N) | 51 9’52” | 51 9’48.5” | 51 9’24” | 49 44’16.3” | 49 51’6” | 49 51’14.8” |
| Longitude (W) | 120 23’18.6” | 120 23’14.1” | 120 24’16” | 120 37’14.7” | 120 34’3” | 120 24’13.1” |
| Elevation (m) | 1479 | 1479 | 1498 | 981 | 1000 | 1001 |
| Perimeter (m) | 750 | 600 | 950 | 415 | 1000 | 460 |

^1^ Big Pantano and Little Pantano are sections of Pantano Lake, which was divided in the summer of 2009.
